# Bereaved Family Members’ Satisfaction with Care during the Last Three Months of Life for People with Advanced Illness

**DOI:** 10.3390/healthcare6040130

**Published:** 2018-11-06

**Authors:** Anna O’Sullivan, Anette Alvariza, Joakim Öhlen, Cecilia Håkanson

**Affiliations:** 1 Department of Health Care Sciences, Palliative Research Centre, Ersta Sköndal Bräcke University College, P.O. Box 11189, SE-100 61 Stockholm, Sweden; anette.alvariza@esh.se; 2 Capio Palliative Care Unit, Dalen Hospital, Åstorpsringen 6, Enskededalen, SE-121 87 Stockholm, Sweden; 3 Institute of Health and Care Sciences, Sahlgrenska Academy at the University of Gothenburg, 41346 Gothenburg, Sweden; joakim.ohlen@fhs.gu.se; 4 Centre for Person-Centered Care, Sahlgrenska Academy at the University of Gothenburg, 40530 Gothenburg, Sweden; 5 The Palliative Centre, Sahlgrenska University Hospital, P.O. Box 457, SE-405 30 Gothenburg, Sweden; 6 Department of Nursing Science, Sophiahemmet University, P.O. Box 5605, SE-114 86 Stockholm, Sweden

**Keywords:** end-of-life care, palliative care, Sweden, quality of health care, proxy measurement

## Abstract

Background: Studies evaluating the end-of-life care for longer periods of illness trajectories and in several care places are currently lacking. This study explored bereaved family members’ satisfaction with care during the last three months of life for people with advanced illness, and associations between satisfaction with care and characteristics of the deceased individuals and their family members. Methods: A cross-sectional survey design was used. The sample was 485 family members of individuals who died at four different hospitals in Sweden. Results: Of the participants, 78.7% rated the overall care as high. For hospice care, 87.1% reported being satisfied, 87% with the hospital care, 72.3% with district/county nurses, 65.4% with nursing homes, 62.1% with specialized home care, and 59.6% with general practitioners (GPs). Family members of deceased persons with cancer were more likely to have a higher satisfaction with the care. A lower satisfaction was more likely if the deceased person had a higher educational attainment and a length of illness before death of one year or longer. Conclusion: The type of care, diagnoses, length of illness, educational attainment, and the relationship between the deceased person and the family member influences the satisfaction with care.

## 1. Introduction

Societal needs for palliative care have increased, due to improved living conditions and enhanced treatments for advanced illnesses, resulting in global ageing populations with a high prevalence of long-term illness [1,2]. Palliative care is a person-centered interdisciplinary approach to care with the aim of improving quality at the end of life and the wellbeing of all patients and their family members facing issues associated with life-limiting illness. The provision of palliative care should, if needed, be provided in all care places and to all patients in need thereof [3], and can accordingly be provided on a general level at home, in nursing homes or hospitals, and through services with palliative expertise (e.g., specialized home care and hospice care) [4]. Central aspects of importance for palliative care are the prevention and relief of suffering, early identification and assessment, treatment of symptoms and other problems (i.e., physical, psychosocial, psychological, and spiritual), communication about end-of-life issues with patients and family members, shared decision-making, and support to family members. Hence, these are also central aspects of importance when evaluating quality in palliative care.

Studies show that individual and socioeconomic factors influence where people are cared for at the end of life and die, and that such factors also may affect the quality of the care within the care places. For example, age is a factor associated with the access to and quality of palliative care, with a higher age resulting in lower access and quality [5,6,7]. Another factor relevant to access to palliative care and adequate end-of-life care interventions is educational attainment, with less well-educated people appearing to be disadvantaged regarding the use of specialist and general palliative care [5,8]. Several studies have also pointed to the diagnosis influencing access to palliative care. For example, people with diseases other than cancer (e.g., COPD (chronic obstructive pulmonary disease), heart disease, and dementia) have poorer access than those with cancer, resulting in less symptom control and less communication about end-of-life issues [9,10,11]. 

Studies about the quality of care in places where most people are cared for during their last months of life, in the Swedish context, are relatively sparse. Assessments of quality have traditionally been based on the health care providers’ perspective, and this has also been the case for palliative care. However, in the past few years, giving a voice to patients’ and family members’ perspectives has been increasingly emphasized [12]. Family members are important sources of knowledge for evaluating care during the last period of life [13,14], and a recognized way to measure the care quality is to evaluate the satisfaction of patients and their family members with the care received. 

Even though the care trajectory in advanced illness usually entails care in several care places, previous studies about family members’ satisfaction with care have often focused only one or two care places. In addition, they usually only focus on the last days/week in life and only specific patient groups (e.g., cancer patients). These studies have shown that several factors are rated as important for a high level of satisfaction with the care provided: information/communication, emotional support, and a healthcare professional pointed out to the family members as overseeing the individual’s care [15,16]. 

The place of care is also important for family satisfaction. Specialized palliative care units have been rated with higher satisfaction than hospitals, nursing homes, and primary health care [17,18,19,20]. However, several studies have also shown that bereaved family members’ satisfaction with care during the terminal phase has been high [15,21,22]. People with advanced illness often have experiences from various care settings during their last period of life. One common care place is the hospital [23]. In studies about care at the end of life in hospitals, shortcomings have been reported regarding palliative care efforts such as symptom relief and timely communication about palliative treatment, death, and dying [6,24,25,26]. Inadequate symptom relief and lack of timely communication have also been reported in studies about palliative care in nursing homes [22,27,28,29]. 

In summary, the relevant international literature indicates that individual and socio-economic factors, diagnosis, and the care place/type of care service are factors that can influence the type of care received, the quality of the care, and the satisfaction thereof. Studies on the evaluation of and satisfaction with care at the end of life do exist for different patient groups in different care places, but the majority present the health care professionals’ perspective of the last days or week of life of the ill person. Hence, there is a need for more comprehensive studies evaluating the care at the end of life for all patient groups in potential need of palliative care, in several care places, for a longer period of the illness trajectory, and based on the perspective of patients and/or their family members. The objectives of this study were to explore bereaved family members’ satisfaction with care during the last three months of life for people with advanced illness, and to investigate associations between satisfaction with care and the characteristics of deceased individuals and family members.

## 2. Materials and Methods

This study had a cross-sectional survey design and was approved by the Regional Ethical board in Stockholm, Sweden: Approval number: 2017/265-31.

### 2.1. Study Context and Sample

Care at the end of life in Sweden can be provided at home, in hospitals, in nursing homes, and in specialized palliative care units (e.g., hospices). The sample consisted of adult bereaved family members of individuals who died in four different hospitals in two Swedish health care regions, between August 2016 and April 2017. The hospitals included from the two regions are general county hospitals. None of the hospitals have specialized high-end care or cancer centers, but all hospitals have oncological clinics. The sample consisted of bereaved family members of individuals who died in a wide range of clinics in the different hospitals. The study participants’ deceased family members had received care from several different types of care services in different care places, besides the care they received before death in one of the hospitals.

The regions chosen for this study were based on a previous population-based place of death study showing regional variations regarding where people are likely to die [30]. The inclusion criteria were: aged 18 years or older (both bereaved family members and deceased individuals), underlying/contributory causes of death (ICD-10 codes) according to the Murtagh et al. [1] model, and death occurring no less than four and no more than twelve months before recruitment to the study. The direct cause of death and the underlying causes of death were based on the physician’s documentation in the patient’s medical records. Murtagh and co-workers’ model includes the following disease categories: HIV/AIDS; Malignant Neoplasm; Alzheimer’s, dementia and senility; Neurodegenerative disease; Heart disease inclusive cerebrovascular disease; Respiratory diseases; Liver disease; and Renal disease. The time period of four to twelve months was based on experiences in previous studies using the VOICES (SF) and the VOICES manual [17,18,31,32]. An additional inclusion criterion was that the deceased individual had an identifiable bereaved family member. The person listed as primary contact in the patient’s data record was invited to participate in the study. 

### 2.2. Recruitment and Data Collection

Of all patients who died in the recruitment hospitals during the study period, 74% (*n* = 1277) met the inclusion criteria. They were identified by hospital administrators based on the inclusion criteria. One health care professional at each hospital (assigned to assist one of the researchers—Anna O’Sullivan) identified the deceased patients’ bereaved family members (*n* = 1277) via the hospital’s patient records. One of the researchers (Anna O’Sullivan) sent written information about the study including the contact information of one of the researchers (Anna O’Sullivan), the VOICES (SF) questionnaire, and a pre-paid return envelope to the included bereaved family members. This written information also indicated that they could withdraw from the study at any time without any explanation or consequence. Furthermore, the participants were assured that the data would be confidential. The participants consented by returning the questionnaire. This was a single-postal survey, meaning that no reminder was sent out. This lack of reminder was for ethical reasons (i.e., being sensitive towards the receivers possibly not wishing to participate). 

#### The VOICES (SF) Questionnaire

The VOICES (SF) questionnaire—Views of Informal Carers–Evaluation of Services (Short Form)—is a questionnaire designed to retrospectively evaluate bereaved family members’ experiences of the quality of care during the last three months of life of an ill family member. VOICES (SF) evaluates the care received in several care places and provides information about both the processes and structures of the care as well as patient outcomes. 

The questionnaire has previously been used for different patient groups and in various healthcare settings, both at population level and in cross-sectional studies, mainly in the United Kingdom, where it was developed [18]. The complete version of VOICES (SF) has been translated and validated into other languages [33,34]. The Swedish culturally adapted and validated version [35] of the questionnaire contains 75 items divided into several domains: Care at home; Care homes; Hospital care; Specialized palliative care units/hospice care. The items are about individual and demographic characteristics (e.g., age, sex, educational attainment, country of birth, relationship to the deceased person), symptom relief, communication, support, collaboration, caregivers’ approaches, and satisfaction with both the care in different care places as well as with the overall care. At the end of the questionnaire, there are three open-ended questions. 

For this study, we used the items regarding care satisfaction and items on individual and demographic characteristics. The items regarding care satisfaction were on a Likert scale of possible responses: excellent, good, fair, poor, and don’t know. One item—“Overall, and taking all services into account, how would you rate his/her care in the last three months of life?”—also had the additional response option “outstanding”. This item is in this study referred to as “overall satisfaction with care”. VOICES (SF) aims to cover as many of the various care places and care services that an individual may have had during the last three months of life, and hence not all items are relevant for all the participating bereaved family members. The items regarding care places or care services not relevant were therefore not answered. The item “overall satisfaction with care” was however answered by all. 

### 2.3. Statistical Analysis

For the quantitative data, explorative descriptive and logistic regression analyses were performed. Descriptive statistical analyses were used to explore the characteristics of the bereaved family members and the deceased individuals, and for the satisfaction with the care in different care places, provided by different caregivers. Multivariable logistic regression analyses were performed to explore associations between overall satisfaction with care and the characteristics of the deceased individuals and their bereaved family members. For these analyses, the forced entry method was initially used, and all co-variables were entered simultaneously. Co-variables were considered to have a significant association with the outcome if *p* < 0.05. The analyses were then also performed as step-wise forward to confirm the significance of the co-variables, by adding the co-variables one by one. The significant co-variables remained significant throughout the whole analysis process, and no other co-variables were significant at any point during the analyses. 

The dependent variable was overall satisfaction with care during the last three months of life, in all care settings. The independent variables were age, sex, and educational attainment for both the deceased individuals and the family members. Additional independent variables were diagnosis; length of illness before death; health care region; and relationship between the deceased person and the family member. Due to the small sample size, the dependent and independent variables were dichotomized and merged to permit logistic regression analyses. The dependent variable was merged into two categories—high or all other—with high including the response categories outstanding/excellent/good, and all other consisting of the categories fair/poor/unknown. This dichotomization of the variable “overall satisfaction” has also been applied in previous studies using VOICES (SF) [22,31,32]. Missing data were excluded from the analyses. The age variable for the deceased individuals was dichotomized into two age categories: “under 85 years old” (<85 years) and “85 years old and over” (>85 years). The age variable for the bereaved family members was dichotomized into two age categories, “18–59 years old” and “60+”. The variable educational attainment was dichotomized into two categories (lower education and higher secondary/higher), and length of illness before death was categorized into “less than 1 year” or “1 year and over”. The variable for cancer/non-cancer was created based on the underlying causes of death and dichotomized, with individuals dying from an illness ICD coded as C00–C99 categorized as cancer and all other diagnoses non-cancer. The non-malignant neoplasms were not represented in the sample. Therefore, the term “cancer” was applied. The variable “relationship between the deceased person and the bereaved family member” was categorical: spouse (including partner), child, and other (e.g., sibling, parent, friend). Two models for analyses were performed, one for associations with the deceased individuals’ characteristics, and one for associations with the bereaved family members’ characteristics. 

Statistical Package for the Social Sciences (SPSS) version 22.0 (IBM Corp., Armonk, NY, USA) was used for all statistical computations. 

### 2.4. Qualitative Descriptive Analysis

In the final part of the questionnaire, three open-ended questions ask the responder if there is anything else they would like to add about the care received, and if there is anything they regard to have been particularly good or bad about the care. For these three questions, a qualitative descriptive analysis was performed [36] to enhance understanding of the quantitative results and to explore aspects of the care that are potentially important for the care satisfaction from the perspective of bereaved family members. The analysis was based on a total of 425 comments made by 220 participants. The number of comments per participant varied between one to three, and some comments comprised several aspects. Initially, all open-ended answers were read closely to get an overview. The answers were then read again to identify and code, with as little interpretation as possible, the statements that represented diverse (positive and negative) experiences of the care, and a quantitative count of the frequencies of statements was made. Finally, the statements were categorized into aspects expressed as particularly positive or negative parts of the care, presented in the Results section. 

## 3. Results

In total, 485 (response rate = 37.9%) bereaved family members participated in the study. The individual characteristics of the non-responding family members were not available. For the deceased individuals linked to the non-responding family members, only age, sex, and diagnosis were available, and there were no differences regarding these from the sample.

### 3.1. Deceased Individuals’ Characteristics

Of the deceased individuals, 50.3% were men, with ages ranging between 40 years old to 90 years or older. The direct causes of death were heart diseases including cerebrovascular diseases (42.5%), respiratory diseases (32.4%), and cancer (20.2%). Heart disease (56.3%) was also the largest underlying cause of death (Table 1). Out of the total 485 deceased individuals, 45.2% received care from district- and county nurses; general practitioners (GPs), 58.7%; specialized home care, 23.8%; hospital care, 90.7%; nursing home care, 28.5%; and hospice care, 16.1%. 

### 3.2. Participant Characteristics—Family Members

Of the 485 participating family members, 70.7% were women. Ages ranged between 18 years old and 90 years or older. Of the participants, 51.4% were children of the deceased person and 34.3% were spouses or partners. See Table 1 for further details. 

### 3.3. Satisfaction with Care

#### 3.3.1. Ratings

**Figure 1:**
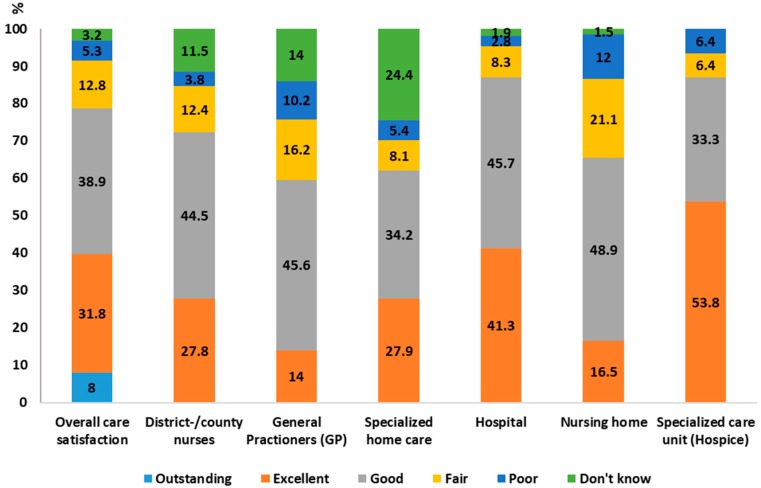
Distribution of bereaved family members’ satisfaction with care, the overall care, and different care places/types of care services. * Percentage of satisfaction is to be seen in proportion of the sample receiving different types of care/services, not as a percentage of the total sample. Missing: Overall care satisfaction 2%; District/county nurses 4.6%; General Practitioners (GPs) 6.7%; Specialized home care 3.5%; Hospital 1.6%; Nursing home 3.6%; Specialized care unit (Hospice) 0%.

Of the participants, 78.7% rated the overall care, taking all care during the last three months into account, as high. For the specific care places/services, 87.1% of the participants reported being satisfied with the care received by the deceased person in a hospice, 87% with the hospital care, 72.3% with district/county nurses, 65.4% with nursing homes, 62.1% with specialized home care, and 59.6% with general practitioners (GPs). See Figure 1 for the detailed distribution of the specific categories of satisfaction. 

#### 3.3.2. Descriptive Responses

The descriptive analysis of the open-ended questions revealed aspects of satisfaction with care related to information and communication, staff performance, and care place/type of care service. Some aspects were reported as particularly positive or as particularly negative, while some aspects were reported as positive when fulfilled and as negative when unmet. 

##### Information and Communication

Aspects of care related to information and communication about the disease, prognosis, and treatments were reported both in the context of particularly positive and particularly negative. Well-functioning communication (i.e., being continuously updated and informed in a sensitive way about the prognosis and about the point at which death was to be expected soon) was reported as positive. A lack of information of a crucial nature, such as prognosis or a rapid decrease in health status and imminent death, were reported as negative aspects for satisfaction with care: “The staff didn’t call us when he got worse during the night. He would have surely appreciated having his close family nearby when he was feeling poorly.”

##### Staff Performance

Satisfaction with care related to staff performance, such as their approach, competence, and commitment was frequently reported. Being treated with respect and dignity by the health care staff was reported as particularly positive. “The staff in the emergency room looked after dad in a professional and nice manner, and us the family members.” The staff’s lack of commitment (e.g., not giving the patient the time needed, being uncaring or uninterested, and revealing a lack of knowledge) was reported as particularly negative. Support from the staff for the family during the illness trajectory, at death, and after the death, as well as the staff’s ability to relieve pain were aspects of satisfaction with care reported as positive when fulfilled, and as negative when unmet.

##### Care Places and Types of Care Services

Frequently reported aspects of care being particularly negative, related to different care places and care services, were poor co-operation between caregivers and internally between the staff, too long a wait in the emergency room, too many emergency room visits or too many care places, as well as the wait to receive or even access certain care: “It took way too long before we got any kind of help, when we finally got him into a short-term nursing home, then he died after two weeks, in a hospital after two days. Alone.”

Specific care places were reported as highly satisfactory and others as not: “The care in the intensive care unit was very good and the contact with and information to us family members was good. The care in the hospital ward was poor. Understaffed, no continuity, very little surveillance and poor information to us family members.” Reported satisfaction with care related to the care places and types of care services also included the availability of the health care provider, the possibility of a single room, and of staying with the dying person. 

### 3.4. Associations between Satisfaction with Care and Co-Variables

Regression analyses for associations between satisfaction with care and co-variables showed significant associations between diagnoses (cancer/non-cancer), length of illness, and educational attainment, as well as the relationship to the deceased person. Bereaved family members that were spouses of deceased individuals were more likely to be satisfied with the care than children or other relatives (OR = 2.86; CI: 1.40–5.80). Furthermore, bereaved family members of deceased individuals with cancer were more likely to express higher satisfaction with the care (OR = 2.10; CI: 1.19–3.73). By contrast, they were more likely to have a lower satisfaction with the overall care if (i) the illness duration (before death) was one year or longer (OR = 1.75; CI: 1.09–2.79); (ii) the deceased person had a higher educational attainment (OR = 2.03; CI: 1.14–3.61); or (iii) the bereaved family member had a higher educational attainment (OR = 2.02; CI 1.08–3.76). There were no significant associations for bereaved family members’ age or sex, nor for the deceased individuals’ age, sex, or the health care region (Table 2).

## 4. Discussion

The results show that the level of satisfaction with care was associated with the bereaved family member’s relationship to the deceased individual and the length of the deceased individual’s illness before death, as well as whether the death was caused by cancer or another illness. The results also show significant associations between satisfaction with care and the deceased individual’s and the bereaved family member’s educational attainment. Satisfaction with care varied for different care places and types of care services, but indicated that the overall satisfaction with the care was relatively high.

The association between level of satisfaction with the care and the bereaved family member’s relationship to the deceased individual was somewhat unexpected, and sheds light on an area that has been the focus of relatively few studies. Ringdal et al. [37] found that children of the deceased individuals were likely to be less satisfied with the care than spouses, in a study measuring bereaved family members’ satisfaction with end of life care. By contrast, Ozcelik et al. [38] found in their study on the satisfaction with care of family members of patients with advanced cancer that the relationship between the bereaved family member and the deceased individual had no significant association for care satisfaction. One possibility is that the relationship has no association with satisfaction of the care when it comes to patients with a cancer diagnosis. It could also be that children of the deceased individuals have been included in the care in different ways compared to spouses, perhaps leading to children being less satisfied with the care. 

The results show that both the length of illness before death and the type of diagnosis influenced the degree of care satisfaction. The importance of diagnosis for access to palliative care has previously been shown in several studies, in which individuals with a cancer diagnosis had greater access to palliative care than non-cancer patients [9,10,39]. This is in line with other studies on access to specialist palliative care for non-cancer patients [40,41,42]. The importance of the care place/type of care services for family satisfaction as well as the quality of the care at the end of life has been shown in previous studies, where specialized palliative care units have been rated with higher satisfaction than hospitals, nursing homes, and primary health care [17,18,19,20,25,26,43,44]. The higher satisfaction with the care received in specialized palliative care units may be explained by the fact that specialized palliative care units are expected to provide palliative care to all their patients, compared to general care units that have both a curative and palliative aim with their care. Hence, the care in specialized palliative care units is also provided by staff with a higher knowledge of and a specialization in palliative care. Studies have shown that several aspects are important to improve palliative care in care places not specialized in palliative care (e.g., knowledge and awareness of palliative care, the staff and time to provide palliative care, as well as the organizational structure for it) [45,46,47]. 

In this study, the bereaved family members with a higher educational attainment were more likely to have a *lower* satisfaction with care, as were family members of the deceased individuals with a higher educational attainment. To the best of our knowledge, this finding has not been confirmed in other published studies. In fact, previous research has more commonly suggested the opposite—that the higher the educational attainment, the greater the access to information and healthcare in general [5,8]. One assumption is that bereaved family members of deceased individuals with a higher educational attainment (or indeed family members with a higher educational attainment themselves) might expect better health care in general. 

The results of the analyses of the answers to the open-ended questions in this study confirmed the importance of the care place/type of care service. Further, the qualitative results showed that communication and information as well as the performance of the staff were valued parts of the care. Several studies have shown the importance of adequate and timely communication regarding the illness, prognosis, and imminent death [20,21,48], as well as the importance of being treated with dignity and respect [20,49,50]. The importance of care place/care services—and differences in care satisfaction related to these—might be especially prominent in this study because of the construction of the VOICES (SF) questionnaire itself. 

### Methodological Considerations

This study’s response rate was at the lower end (37.9%), and could potentially have been improved by using reminders and repeated mail outs. However, this was not done for ethical reasons —to respect and avoid upsetting participants who did not wish to take part. Some of the deceased persons had several different care places and care services during the last three months of life. Unfortunately, we do not know the reason for care admissions or the length of care in each care place, or if anything particularly crucial occurred in a certain care place, because this information is not included in the questionnaire items. Hence, we do not know if and how this affected the overall satisfaction with care during the last three months, and this must be regarded as a limitation of the study results. Hence, the VOICES (SF) questionnaire has the benefit of enabling the evaluation of most care services received during the last three months of life, but it does have limits regarding the possibilities for analyses of the answers for the care trajectories, since it does not provide information on the length, reason for, or order of the different care events. 

None of the participants have contacted the researchers to report any negative reactions towards the request for participation or towards the questionnaire itself. A few stated that they did not wish to participate because they did not feel well or because they were still grieving, whereas others very much appreciated the opportunity to share their experiences and found answering the questions beneficial for their bereavement. 

The results from this study can make no claim of generalization on a population level, since the sample consists of the bereaved family members of individuals who died in four hospitals in two Swedish health care regions and should be understood in that context. A further limitation is that the sample consisted mainly of native Swedes, and the very small part of the sample with other origin was mainly from other Scandinavian countries, which is not representative of the pluralism of Sweden. Still, the study does provide new and important knowledge about bereaved family members’ satisfaction with the care received during the last three months of life.

## 5. Conclusions

This study shows that most of the bereaved family members reported being highly satisfied with the care received during the last three months of life. However, almost one-fifth (*n* = 86) of the participants rated their satisfaction with the overall care as low. Factors seemingly influencing the bereaved family members’ care satisfaction were the relationship to the deceased person, a cancer diagnosis, educational attainment, and the length of illness prior to death. The results also show that the bereaved family members had the highest satisfaction with the care provided in specialized palliative care units and the lowest with the care from general practitioners. Hence, it is plausible that the type of care services and care place are important for the satisfaction with the care. However, further studies are needed to explore in more depth the quality of and satisfaction with care in different care places and to further investigate the factors that are crucial for a high/low satisfaction with care at the end of life. 

## Figures and Tables

**Table 1:** Characteristics of deceased individuals and family members.

Characteristics	Deceased Individuals		Family Members	
	% ^a^	*n*	% ^a^	*n*
Sex (Missing = x/485)	-	(0/485)	-	(0/485)
Male	50.3	(244)	29.3	(142)
Female	49.7	(241)	70.7	(343)
Age (Missing = x/485)	-	(1/485)	-	(8/485)
18–29	-	-	0.8	(4)
30–39	-	-	1.6	(8)
40–49	1.2	(6)	6.8	(33)
50–59	2.3	(11)	22.3	(108)
60–69	8.9	(43)	31.3	(152)
70–79	23.1	(112)	22.9	(111)
80–89	36.7	(178)	11.3	(55)
90+	27.6	(134)	1.2	(6)
Educational attainment (Missing = x/485)	-	(5/485)	-	(3/485)
Lower secondary education	72.4	(351)	29.5	(143)
Higher secondary education	11.1	(54)	30.5	(148)
Higher education	15.5	(75)	39.4	(191)
Direct cause of death ^b^	-	-	-	-
Cancer	20.2	(66)	-	-
Heart diseases (incl. cerebrovascular)	42.5	(139)	-	-
Alzheimer’s	0	(0)	-	-
Respiratory diseases	32.4	(106)	-	-
Renal diseases	3.1	(10)	-	-
Neurodegenerative diseases	0.6	(2)	-	-
Liver diseases	1.2	(4)	-	-
Underlying cause of death 1 ^b^	-	-	-	-
Cancer	15.8	(64)	-	-
Heart diseases (incl. cerebrovascular)	56.3	(228)	-	-
Alzheimer’s	1.0	(4)	-	-
Respiratory diseases	15.1	(61)	-	-
Renal diseases	9.4	(38)	-	-
Neurodegenerative diseases	1.0	(4)	-	-
Liver diseases	1.5	(6)	-	-
Underlying cause of death 2 ^b^	-	-	-	-
Cancer	15.2	(52)	-	-
Heart diseases (incl. cerebrovascular)	64.3	(220)	-	-
Alzheimer’s	3.5	(12)	-	-
Respiratory diseases	10.2	(35)	-	-
Renal diseases	4.4	(15)	-	-
Neurodegenerative diseases	0.9	(3)	-	-
Liver diseases	1.2	(4)	-	-
HIV/Aids	0.3	(1)	-	-
Length of illness before death (Missing = x/485)	-	(6/485)	-	-
Sudden	5.4	(26)	-	-
<24 h	2.1	(10)	-	-
>24 h–1 week	10.7	(52)	-	-
>1 week–1 month	13.0	(63)	-	-
>1 month–6 months	14.8	(72)	-	-
>6 months–1 year	10.3	(50)	-	-
1 year or more	42.5	(206)	-	-
Relationship (Missing = x/485)	-	(4/485)	-	-
Spouse	34.5	(166)	-	-
Child	51.8	(249)	-	-
Other ^c^	13.7	(66)	-	-

^a^ Column percentage displayed; ^b^ Underlying causes of death according to Murtagh’s (2014) model for potential palliative care needs; ^c^ Other = e.g., sibling, friend, parent.

**Table 2:** Associations between the deceased individuals’ and bereaved family members’ characteristics, and the bereaved family members’ overall satisfaction with care during the last three months of life in all care places.

Co-Variables	Overall High Satisfaction OR ^a^ 95% CI ^b^	*p*-Value ^c^
Deceased individuals	-	-
Sex	-	-
Male	1 ^d^	
Female	0.98 (0.62–1.56)	0.95
Age	-	-
<85	1	-
>85	1.54 (0.94–2.51)	0.08
Underlying cause of death	-	-
Non-cancer	1	-
Cancer	2.10 (1.19–3.73)	0.01
Length of illness before death	-	-
Less than 1 year	1	-
1 year or more	0.57 (0.35–0.91)	0.01
Health Care region	-	-
Southeast	1	-
Stockholm	1.29 (0.81–2.07)	0.27
Bereaved family members	-	-
Sex	-	-
Male	1	-
Female	0.84 (0.49–1.43)	0.53
Age	-	-
18–59	1	-
60+	1.05 (0.37–1.93)	0.85
Relationship	-	-
Spouse	2.86 (1.40–5.80)	0.00
Child	1.98 (1.07–3.69)	0.03
Other ^e^	1	-
Educational attainment	-	-
Lower/elementary	1	-
Higher secondary/higher	0.49 (0.26–0.92)	0.02

^a^ Odds ratio; ^b^ Confidence interval; ^c^ Regression coefficient significant if *p* < 0.05 for Chi-square; ^d^ Categories with the value 1 are the reference category; ^e^ Other = e.g., sibling, friend, parent.
